# Functional insights from proteome-wide structural modeling of *Treponema pallidum* subspecies *pallidum*, the causative agent of syphilis

**DOI:** 10.1186/s12900-018-0086-3

**Published:** 2018-05-16

**Authors:** Simon Houston, Karen Vivien Lithgow, Kara Krista Osbak, Chris Richard Kenyon, Caroline E. Cameron

**Affiliations:** 1 Department of Biochemistry and Microbiology, University of Victoria, Victoria, British Columbia Canada; 2 HIV/STI Unit, Institute of Tropical Medicine, Antwerp, Belgium; 3 Division of Infectious Diseases and HIV Medicine, University of Cape Town, Cape Town, South Africa

**Keywords:** *Treponema pallidum*, Syphilis, Proteome, Structural modeling, Functional annotation, Virulence factors

## Abstract

**Background:**

Syphilis continues to be a major global health threat with 11 million new infections each year, and a global burden of 36 million cases. The causative agent of syphilis, *Treponema pallidum* subspecies *pallidum*, is a highly virulent bacterium, however the molecular mechanisms underlying *T. pallidum* pathogenesis remain to be definitively identified. This is due to the fact that *T. pallidum* is currently uncultivatable, inherently fragile and thus difficult to work with, and phylogenetically distinct with no conventional virulence factor homologs found in other pathogens. In fact, approximately 30% of its predicted protein-coding genes have no known orthologs or assigned functions. Here we employed a structural bioinformatics approach using Phyre2-based tertiary structure modeling to improve our understanding of *T. pallidum* protein function on a proteome-wide scale.

**Results:**

Phyre2-based tertiary structure modeling generated high-confidence predictions for 80% of the *T. pallidum* proteome (780/978 predicted proteins). Tertiary structure modeling also inferred the same function as primary structure-based annotations from genome sequencing pipelines for 525/605 proteins (87%), which represents 54% (525/978) of all *T. pallidum* proteins. Of the 175 *T. pallidum* proteins modeled with high confidence that were not assigned functions in the previously annotated published proteome, 167 (95%) were able to be assigned predicted functions. Twenty-one of the 175 hypothetical proteins modeled with high confidence were also predicted to exhibit significant structural similarity with proteins experimentally confirmed to be required for virulence in other pathogens.

**Conclusions:**

Phyre2-based structural modeling is a powerful bioinformatics tool that has provided insight into the potential structure and function of the majority of *T. pallidum* proteins and helped validate the primary structure-based annotation of more than 50% of all *T. pallidum* proteins with high confidence. This work represents the first *T. pallidum* proteome-wide structural modeling study and is one of few studies to apply this approach for the functional annotation of a whole proteome.

**Electronic supplementary material:**

The online version of this article (10.1186/s12900-018-0086-3) contains supplementary material, which is available to authorized users.

## Background

The medically important spirochete bacterium *Treponema pallidum* subspecies *pallidum* is the causative agent of syphilis. Syphilis is a chronic, multi-stage, systemic disease with three major modes of transmission; via sexual contact, exposure to an infectious lesion, and in utero. Although syphilis can be readily treated with penicillin, approximately 11 million new infections occur each year, contributing to a global burden estimated to comprise 36 million cases [1]. Congenital syphilis, which results in spontaneous abortion, stillbirth, postpartum death, or newborn malformations, is also a major global health issue with an estimated 1.4 million pregnant women infected with syphilis each year [2]. In addition, it has been well established that symptomatic syphilis infection increases the risk of HIV transmission and acquisition two- to five-fold [3]. The global public health threat posed by syphilis emphasizes the need to gain a better understanding of the molecular mechanisms underlying *T. pallidum* pathogenesis, and in particular, the identification of suitable protein targets for vaccine design and diagnostic test applications.

*Treponema pallidum* is a highly virulent bacterium and is one of the most invasive pathogens known. Following infection, it rapidly invades the host tissue barrier and disseminates to distant tissues and organs via the circulatory system [4–6]. It is also one of the few pathogens capable of traversing both the placental and blood-brain barriers [4]. Although 19 years have passed since the publication of the first *T. pallidum* complete genome sequence [7], virulence factors remain to be definitively identified. This is primarily due to four reasons. First, *T. pallidum* is an obligate human pathogen that is unable to be cultured continuously in vitro or genetically manipulated. The inability to culture *T. pallidum* is consistent with its reduced genome, which is comprised of 978 predicted protein-coding genes [8], and the corresponding absence of key biosynthetic pathways. Second, the bacterium is inherently fragile due to the presence of a unique outer membrane ultrastructure with few integral outer membrane proteins and an unusually thin peptidoglycan layer that is more distal to the outer membrane compared to conventional Gram-negative bacteria [9, 10], which renders laboratory manipulation challenging. Third, *T. pallidum* is a phylogenetically distinct bacterium, highlighted by the fact that almost 30% of predicted protein-coding genes still have no known orthologs or assigned functions [7, 8]. Finally, genome sequencing of *T. pallidum* identified very few conventional virulence factor candidates [7, 8].

Protein structure determination informs protein function and thus can be used to overcome some of the knowledge acquisition roadblocks stemming from experimental limitations associated with *T. pallidum* research. The three-dimensional structure of a protein determines its function, and protein structure is oftentimes more conserved than protein sequence [11]. Hence, the solving of high-resolution protein structures has the potential to provide insight into the molecular mechanisms that mediate *T. pallidum* pathogenesis through delineation of structure-function relationships. However, to date only 18 unique protein structures from the 978 *T. pallidum* predicted protein-coding genes have been determined and deposited in the Research Collaboratory for Structural Bioinformatics Protein Data Bank [12, 13], representing only 1.8% of the proteome [14–31]. The analysis of whole proteome sequences using computational methodologies for protein tertiary structure modeling provides an alternative structural bioinformatics-based approach for improving our understanding of potential protein functions on a proteome-wide scale. Phyre2 [32] is a widely-used protein tertiary structure modeling server that consistently ranks among the top-scoring protein tertiary modeling servers in critical assessment of protein structure prediction (CASP) trials [33, 34]. In addition, unlike other molecular modeling servers, Phyre2 is capable of rapidly modeling multiple proteins which makes it particularly well suited for analyzing whole proteomes. However, to date Phyre2 has only been employed for the structural and functional annotation of a limited number of whole proteomes [35–37].

In the current study, we used a Phyre2-based structural bioinformatics approach for molecular modeling and functional inference of the whole *T. pallidum* proteome. This methodology generated high confidence tertiary structure model predictions that provide novel insight into the structure and function of *T. pallidum* proteins with no known orthologs or assigned functions that may play important roles in the unique structural and virulence properties of *T. pallidum*. Furthermore, we found a high level of protein functional annotation agreement between our molecular modeling-based approach and genome sequencing pipelines that have relied primarily on amino acid sequence homology comparisons. These findings provide support for the effectiveness of structural modeling as a viable approach for predicting protein function on a proteome-wide scale, and suggest it may also serve as a valuable complementary tool in genome annotation pipelines. Finally, our structural bioinformatics approach also identified several potential *T. pallidum* virulence factors that represent promising targets for future experimental studies.

## Methods

### *Treponema pallidum* subspecies *pallidum* genome sequence

The genome of *T. pallidum* subspecies *pallidum* (Nichols) was recently re-sequenced, which resulted in improved sequence accuracy and functional annotation [8]. This sequencing project used next-generation sequencing techniques which resulted in the identification of 978 predicted protein-coding genes. In the present study, we used the re-sequenced *T. pallidum* (Nichols) genome sequence (GenBank Accession Number CP004010.2) [8] for all tertiary structure modeling and analyses.

### Whole proteome modeling of *T. pallidum* using Phyre2

Phyre2 is a web-based bioinformatics server for the prediction of protein structure, based on the principles of homology-based modeling [32, 38]. For each protein sequence to be modeled, there are four stages. The first stage involves the identification of homologous sequences and the generation of a multiple-sequence alignment (MSA). This is achieved by using the sequence alignment search tool HHBlits [39] to scan the query protein sequence against a non-redundant database which generates the protein’s sequence profile. PSIPRED [40] uses the MSA to predict secondary structure, and both the MSA profile and secondary structure predictions are then combined into a query hidden Markov model (HMM). In stage two, the query protein HMM is scanned against a fold library comprised of HMMs of known structure using the alignment algorithm, HHSearch [41]. The highest scoring alignments are used to generate three-dimensional backbone models. Stages three and four involve modeling of loops and fitting of side-chains using the R3 protocol [42] to generate the final three-dimensional structural model, respectively.

**Fig. 1:**
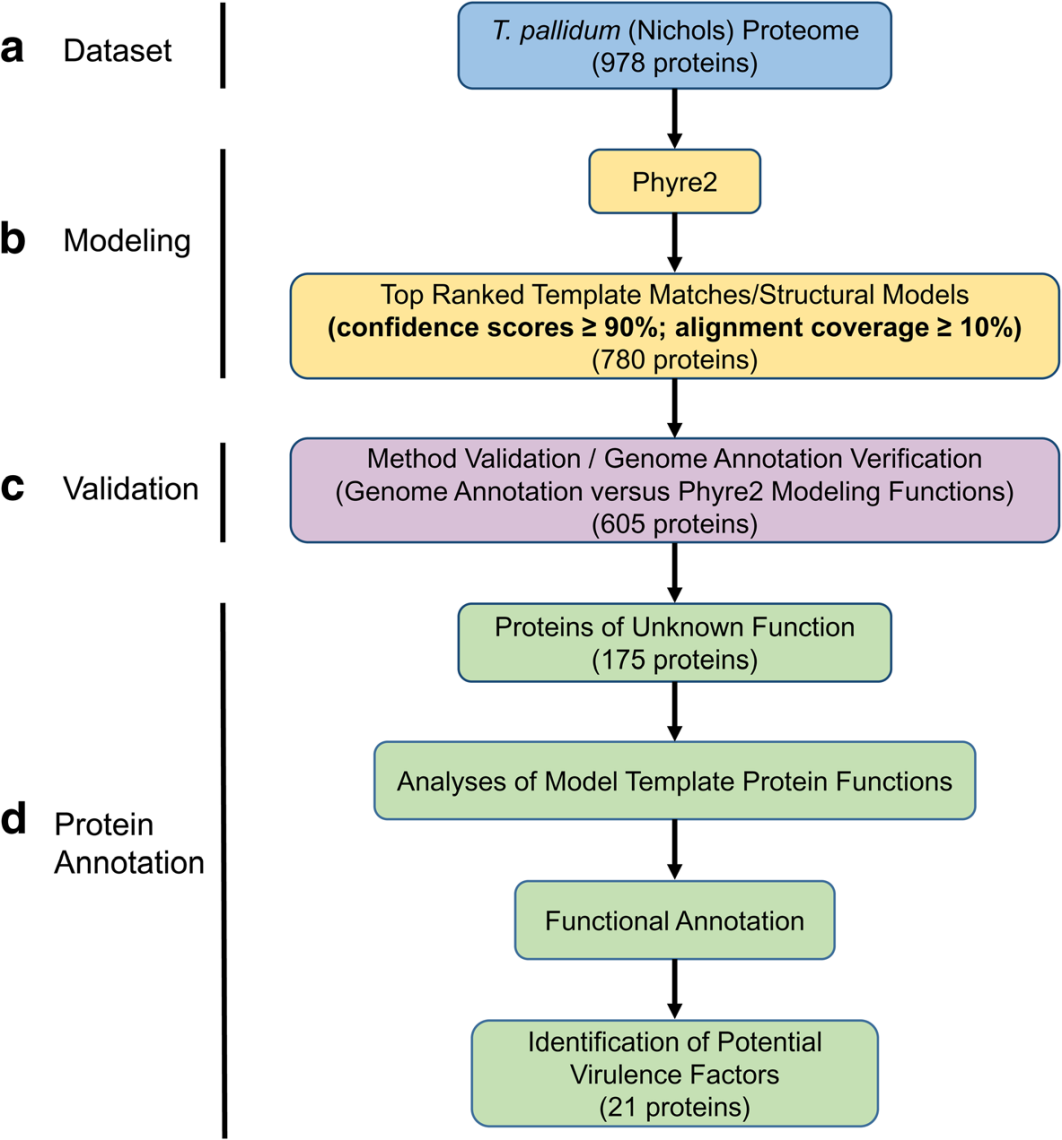
Pipeline for proteome-wide tertiary structure modeling of *T. pallidum*. **a** Dataset: All 978 proteins from *T. pallidum* subspecies pallidum (Nichols) were used for whole proteome tertiary structure modeling and functional predictions. **b** Modeling: Complete amino acids sequences corresponding to the 978 protein-coding genes were submitted in “batch-mode” to the protein tertiary structure modeling server, Phyre2, and the 20 top-ranked template structural matches for each protein were obtained. Only those *T. pallidum* proteins that were modeled with a Phyre2 confidence score of at least 90% and alignment coverage of at least 10% were analyzed further. **c** Validation: To help validate our approach and increase confidence in previous genomic annotations, the predicted functions (based on tertiary structure model template information) of 605 *T. pallidum* proteins were compared with their corresponding functional annotations derived from genome sequencing. Proteins were then categorized as having either the same, related, or different functions. **d** To gain insight into the potential function(s) of 175 uncharacterized proteins, and for the identification of potential virulence factors, the functions of the 20 top-ranked tertiary structure model templates for each protein were analyzed using the same confidence and alignment coverage cut-off scores as described above

The flow diagram shown in Fig. 1 outlines all major steps that comprised the Phyre2-based tertiary structure modeling pipeline of the *T. pallidum* proteome. In order to predict the three-dimensional structure of all *T. pallidum* proteins, and as the first step in gaining functional insights from proteome-wide structural modeling of *T. pallidum*, amino acid sequences corresponding to the 978 protein-coding genes identified by the recent re-sequencing of *T. pallidum* [8] were submitted in “batch mode” (maximum of 100 sequences per submission) to the protein fold recognition server, Phyre2 [32] (Fig. 1a). For each of the 978 *T. pallidum* protein sequences, the 20 top-ranked template structural matches were obtained, corresponding to 19,560 templates with associated predicted models (Fig. 1b). Ranking is based on the number of amino acid residues that were aligned and the quality of the alignment. Alignment quality is based on the level of secondary structure similarities between the query sequence and template, the amount of insertions and deletions between the two sequences, and the similarity of amino acid probability distributions at each position of the query and templates sequences [32]. Detailed template information relating to each ranked model was also obtained. These included a text description of the protein template including PDB descriptions (classification, header/molecule, and structure title fields) and the template PDB code. A confidence score (probability that the protein query/template match is a true homology), alignment coverage (coverage of the query protein sequence within the aligned region), and sequence identity (amino acid identity between the query and template protein sequences within the aligned region) were also obtained (all range from 0 to 100%). All coordinate files for each model in PDB format were downloaded for visualization and manipulation of each predicted model in the protein structure viewing/modeling program, UCSF Chimera [43, 44], and the corresponding Phyre2 batch mode results were incorporated into Microsoft Excel spreadsheets for data analyses (for raw unedited Phyre2 data corresponding to 19,560 templates used for whole proteome modeling, refer to Additional file 1: Table S1 [part 1]).

### Identification of predicted *T. pallidum* protein tertiary structures modeled with high confidence

A structural model and template match with a confidence score of 90% or greater is considered highly reliable. This indicates the query protein is very likely to adopt the overall predicted fold and that the protein core is modeled at high accuracy (2–4 Å root-mean-square deviation from the true protein structure) [32]. Therefore, for our analyses we set the minimum cut-off for confidence scores at equal to or greater than 90% (Fig. 1b).

### Rationale for alignment coverage minimum cut-off value

The average *T. pallidum* protein size is approximately 340 amino acids [45]. However, several structures of protein functional domains from other organisms as small as 30–40 amino acids have been solved and deposited in the RCSB PDB. Thus, in order to further increase the overall confidence in the modeling without omitting small potential functional domains (~ 30–40 amino acids), we set the minimum cut-off value for alignment coverage at 10% or greater (Fig. 1b). Sequence identity was not taken into account to increase model confidence at the whole proteome-modeling stage of the analyses. This was due to the fact that *T. pallidum* is genetically distinct from all other bacteria, including other treponemal species, and this study was focused on using tertiary structure prediction to decipher potential protein function in the absence of high amino acid sequence homology.

### Method validation: Determining the level of correlation between *T. pallidum* genome sequence annotation and Phyre2-based annotation

To determine how well our Phyre2 analysis correlates with the genome sequence functional annotation, the predicted functions (derived from the model templates) of *T. pallidum* proteins modeled with high confidence (all template matches for each protein with ≥90% confidence and ≥ 10% alignment coverage) in the current study were compared with functional annotations derived from genome sequencing [8] (Fig. 1c). This analysis compared the protein functions inferred from Phyre2 modeling using (1) only the highest confidence tertiary structure model template function (proteins were assigned a function based solely on the single top-ranking template) and (2) all confident tertiary model protein template functions from the top 20 ranking models (proteins were assigned a function based on all template matches for each protein that generated models with ≥90% confidence and ≥ 10% alignment coverage). Proteins were categorized as having either the same function as the published genome annotations, related functions (same PDB functional group classification and/or related PDB molecule/template function), or alternative functions (including unknown functions).

### Functional annotation of uncharacterized *T. pallidum* proteins

To gain insight into the potential function of uncharacterized proteins, we analyzed the functions of the 20 top-ranked template structural matches that Phyre2 used for modeling each protein with confidence and alignment cut-off values as described above (Fig. 1d).

## Results

### Distribution of structure prediction confidence and coverage scores from *T. pallidum* proteome-wide structural modeling

**Fig. 2:**
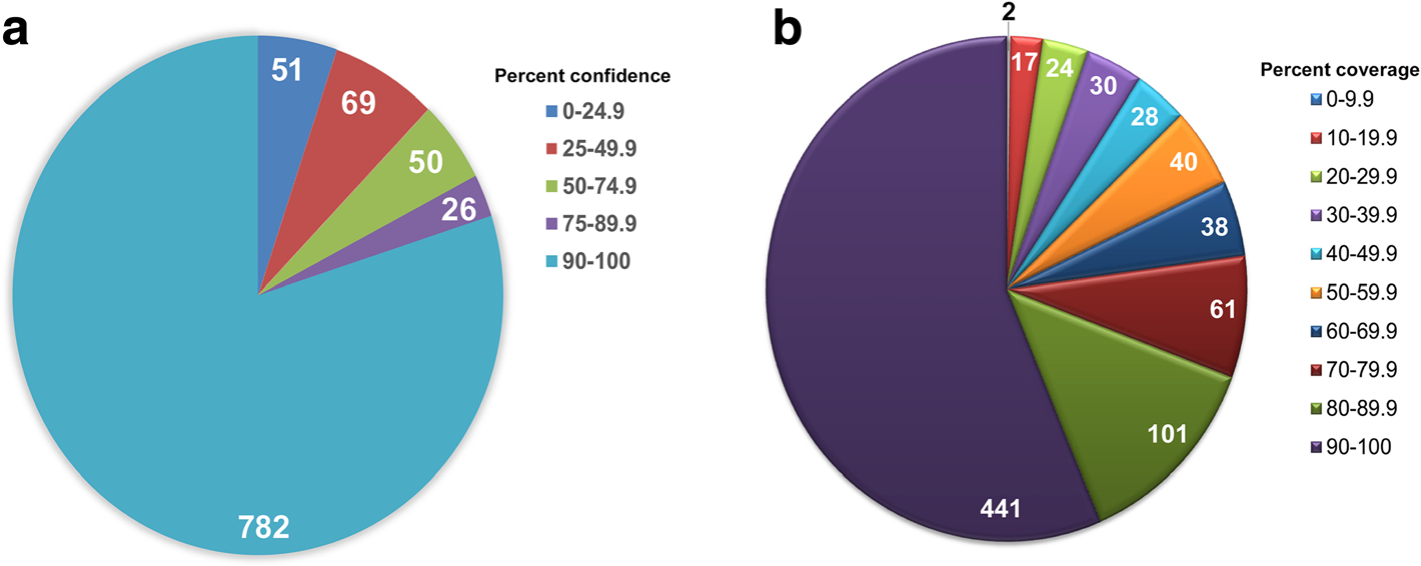
Distribution of confidence and coverage scores from *T. pallidum* proteome-wide structural modeling. Amino acid sequences of 978 protein-coding genes from *T. pallidum* subspecies *pallidum* were submitted to Phyre2 for structural modeling. **a** Pie chart indicating the distribution of the number of *T. pallidum* proteins with tertiary structure model predictions within five Phyre2 confidence score ranges. A confidence score of at least 90% was used for all subsequent analyses. **b** Pie chart indicating the distribution of the number of *T. pallidum* proteins modeled by Phyre2 with ≥90% confidence scores within 10% alignment coverage categories (0–100%). An alignment coverage of at least 10% was used for all subsequent analyses

The first step in our *T. pallidum* whole proteome modeling pipeline was to identify all proteins modeled by Phyre2 that have confidence scores of at least 90% (Fig. 1a and b). Out of 978 *T. pallidum* protein sequences submitted, 782 (80%) were modeled with a confidence score equal to or greater than our minimum cut-off value (Additional file 1: Table S1 and Fig. 2a). Of the 196 *T. pallidum* proteins that were modeled with confidence scores below our minimum cut-off value, 150 (77%) were annotated in the published *T. pallidum* proteome [8] as “hypothetical” or “putative membrane proteins” (Additional file 2: Table S2). The remaining 46 (23%) low-confidence models had functional annotations in the published *T. pallidum* proteome based on amino acid sequence homology (Additional file 3: Table S3). When we analyzed the physicochemical properties of the 46 proteins with low confidence models, wide value ranges were observed for each property with values similar to those previously reported for all *T. pallidum* proteins [7] (Additional file 3: Table S3). Nine of the 46 low-confidence models are annotated in the published proteome [8] as flagellar proteins, three are annotated as type 3 secretion system (T3SS)/injectisome proteins, eight are annotated as transport proteins, 16 are annotated with miscellaneous functions, and 10 belong to the *Treponema pallidum* repeat (Tpr) protein family [46] (Additional file 3: Table S3). Subcellular localization analyses predicted approximately 50% of the proteins to be localized to the inner membrane with 27–35 also predicted to contain transmembrane helices (Additional file 3: Table S3), which is indicative of an inner membrane locale.

To increase confidence in our modeling pipeline, we set the minimum alignment coverage cut-off value at 10% or greater. A total of 780/782 *T. pallidum* proteins that were modeled with at least 90% confidence also had alignment coverage of at least 10%, representing 80% of the *T. pallidum* proteome, (Fig. 2b). All 780 *T. pallidum* proteins modeled with at least 90% confidence and 10% alignment coverage together with all corresponding model details are listed in Additional file 1: Table S1 (part 2). Furthermore, 681/782 (87%) high confidence-scoring models/templates were based on alignments that covered at least 50% of the *T. pallidum* protein, representing 70% (681/978) of all *T. pallidum* predicted protein-coding genes (Fig. 2b). Taken together, these results show that Phyre2-based whole proteome analysis is capable of predicting tertiary structure models with high confidence and biologically relevant alignment coverage for the majority of *T. pallidum* proteins.

### Comparison of *T. pallidum* primary structure- and tertiary structure-based proteome annotations

**Fig. 3:**
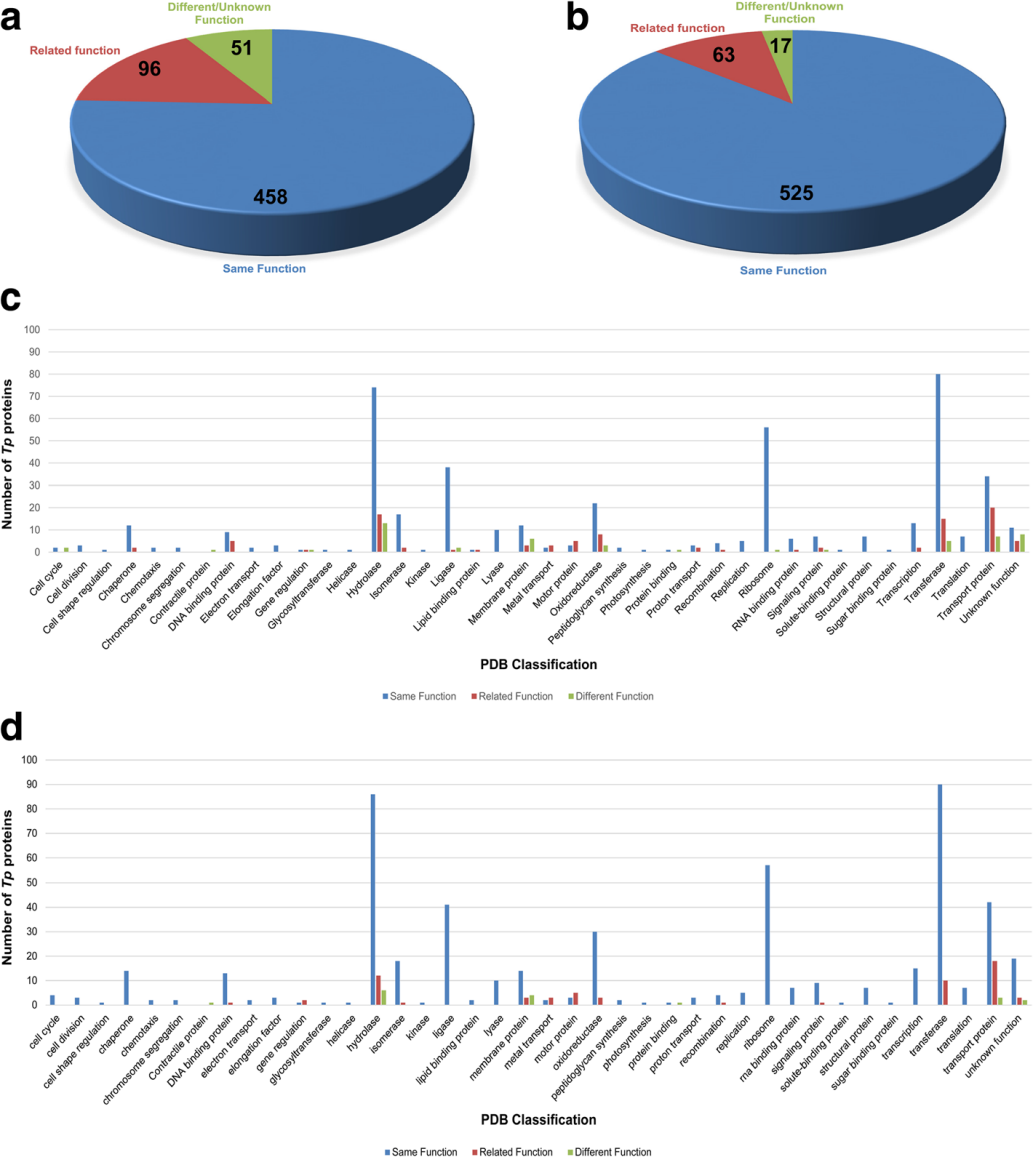
Comparison of *T. pallidum* primary and tertiary structure annotations. Predicted functions of 605 *T. pallidum* proteins derived from tertiary structure models with high confidence were compared with functional annotations from genome sequencing. **a** Distribution of *T. pallidum* proteins modeled by Phyre2 (≥ 90% confidence, ≥10% alignment coverage) predicted to have the same, related (same PDB functional group classification and/or related PDB molecule/template function), or different functions (including unknown functions) compared to the published *T. pallidum* (Nichols) genome annotations. This analysis only used the top-ranking tertiary structure model protein template function. **b** Distribution of *T. pallidum* proteins as outlined above after genome annotated functions were compared to all confident (≥90% confidence, ≥10% coverage alignment) top 20-ranking templates used to model each protein. A functional match was assigned when the genome annotated function matched at least one protein tertiary structure model template function. **c** Distribution of *T. pallidum* proteins as outlined above according to their PDB functional classification using the top-ranking template only, or (**d**) using all confident tertiary structure model top 20-ranking templates for each protein function comparison. It should be noted that in (**c**) and (**d**), protein templates used to model a small number of *T. pallidum* proteins in the current study were categorized as “unknown function” by the PDB classification system. However, these proteins were ascribed functions in the current study based on their PDB molecule function annotation and/or PDB structure title which allowed for comparisons with their genomic protein annotations

Most modern genome annotation pipelines are multi-level processes, however they still rely on inferring protein function from primary structure homology. Six hundred and five of the 780 *T. pallidum* proteins modeled with high confidence and alignment coverage in the current study were previously annotated with specific functions in the published *T. pallidum* genome. To determine the level of annotation agreement between the two methods, we compared the 605 Phyre2-predicted protein functions (derived from their model template functions) with the corresponding published protein annotations [8] (Fig. 1c). When comparing only the top-ranked Phyre2 protein template match for predicting function, 458/605 (76%) were assigned the same function as the published genomic annotations (Fig. 3a). Genome annotations were also compared to all confident (≥90% confidence and ≥ 10% coverage alignment) top 20-ranking templates used to model each protein and a functional match was assigned when the genome annotation matched at least one protein template function. Using this comparison, 525/605 (87%) were assigned the same functions with respect to the published genome (Fig. 3b). Seventeen proteins were also confidently modeled on protein templates whose functions differed from the corresponding genomic annotations (Fig. 3b). This was most likely due to the fact that solved structures of these 17 proteins have not yet been deposited in the PDB. All proteins with the same, related, or different functions were categorized according to their PDB functional classification (Fig. 3c [genome annotation versus top-ranking template only comparison] and Fig. 3d [genome annotation versus all confident top 20-ranking templates for comparison]) and are listed in Additional file 4**:** Table S4 (top matching template only comparison) and Additional file 5: Table S5 (all confident matching templates used for comparison).

Importantly, when we also compared our molecular modeling-based protein function predictions with *T. pallidum* proteins that have been characterized experimentally, we observed a high degree of correlation at both the structural and functional levels. For this analysis, we focused on 11 functionally-uncharacterized proteins that were successfully assigned specific functions (TPANIC_0163 [TroA] [26], TPANIC_0298 [RfuA] [30], TPANIC_0319 [PnrA] [21], TPANIC_0435 [Tp17] [24], TPANIC_0574 [Tp47] [16], TPANIC_0655 [TpPotD] [23], TPANIC_0796 [Ftp] [27], TPANIC_0821 [Tp32] [18], TPANIC_0956 [TatT] [29], TPANIC_0957 [TatP(T)] [29], TPANIC_0971 [Tp34] [22]) using a structure-to-function approach comprised of three major stages; (1) X-ray crystallography that determined the high resolution structures of the 11 functionally-uncharacterized treponemal proteins, (2) protein structure analyses for hypothesizing potential protein functions, and (3) biochemical characterization of the proteins to confirm or reject the hypotheses. When comparing only the top-ranked Phyre2 protein model and template match for predicting function, ten of the 11 *T. pallidum* proteins were modeled on the functionally-characterized proteins listed above (Additional file 1: Table S1 [part 1]). The exception was TPANIC_0435 (Tp17) which was modeled with highest confidence on a putative lipoprotein from the proteobacterium, *Shewanella oneidensis* (100% confidence, 71% alignment coverage). However, this protein was also modeled with a very similar high level of confidence against the Tp17 structural template (99.7% confidence, 76% alignment coverage) (Additional file 1: Table S1 [part 1]). A contributing factor that may partially account for this discrepancy is the fact that the protein structure of the top-ranking model from *S. oneidensis* was solved at greater resolution than the *T. pallidum* Tp17 protein (1.42 Å versus 2.4 Å). It should also be noted that Phyre2 indicated that the Tp17 model template exhibited 100% sequence identity to the query *T. pallidum* protein TPANIC_0435. Although this study focused on using tertiary structure modeling to predict protein function in the absence of high amino acid sequence homology, this finding would need to be taken into consideration when deciding which model is likely more accurate. When comparing all remaining confident (≥90% confidence and ≥ 10% alignment coverage) top 20-ranking templates used to model each of the 11 proteins (ranks 2–20), 11/11 were modeled against protein templates with the same, or very closely related, functions as the 11 functionally-characterized proteins listed above (Additional file 1: Table S1 [part 1]). Together, these findings suggest tertiary structure modeling may serve as a complementary tool to genome sequencing/annotation pipelines for assigning potential protein functions on a proteome-wide scale.

### Predicted annotation of *T. pallidum* proteins of unknown function

In the current study, 175 of the 780 *T. pallidum* proteins modeled with high confidence/alignment coverage were annotated in the published proteome as “hypothetical” proteins (142/175), putative membrane proteins (27/175), or putative outer membrane proteins (6/175) [8] (Additional file 6**:** Table S6). DNA microarray-based analysis of the *T. pallidum* transcriptome following experimental rabbit infection [45] showed that almost all of the genes encoding these uncharacterized proteins (173/175) are expressed at the transcription level (Additional file 6: Table S6). In fact, their mean transcript expression level (cDNA/DNA ratio = 1.34) was found to be higher than the mean transcript level (cDNA/DNA ratio = 1.0) of all *T. pallidum* genes in the array (Additional file 6: Table S6). Furthermore, two mass spectrometry-based proteomics studies also detected peptides from 107 of the 175 uncharacterized proteins during rabbit infection [47, 48] (Additional file 6: Table S6).

**Fig. 4:**
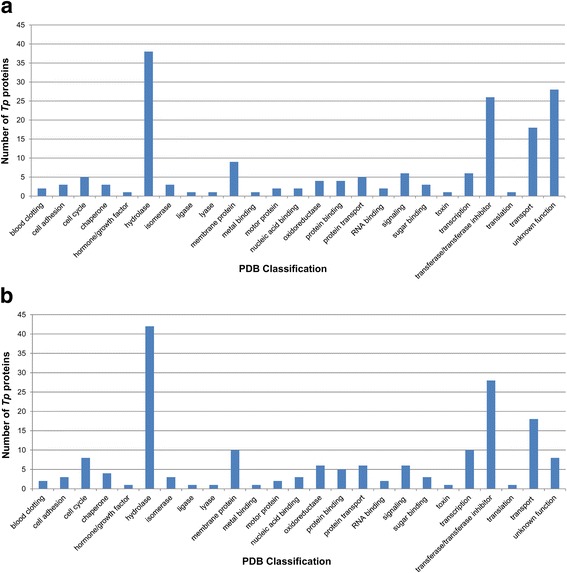
Functional annotation of *T. pallidum* hypothetical proteins using Phyre2. The potential function of 175 uncharacterized proteins were predicted by analyzing the functions of the template proteins Phyre2 used for tertiary structure modeling. **a** Distribution of PDB functional classes within the *T. pallidum* proteome based on Phyre2 modeling using the top-ranking tertiary structure model template only, or (**b**) by comparing the genome annotated functions to all confident (≥90% confidence, ≥10% coverage alignment) tertiary structure model top 20-ranking templates that were used to model each protein

Although the potential functions of some of these 175 proteins of unknown function have been investigated within laboratory settings [17, 49–53] (Additional file 6: Table S6), we decided to focus on all of these proteins in order to gain further insight into the unusual biology of *T. pallidum* (Fig. 1d). When analyzed using only the highest ranking confident tertiary structure model protein template as an indication of protein function, 147/175 (84%) were assigned potential functions (≥90% confidence and ≥ 10% alignment coverage) (Fig. 4a and Additional file 6: Table S6). The 28 uncharacterized proteins that were modeled against structures classified by PDB as “unknown function” are listed in Additional file 7: Table S7. Of the 175 *T. pallidum* proteins of unknown function, 38 (21.7%) were modeled against hydrolases using PDB functional classifications (Fig. 4a). As expected from this broad classification system, this group included proteins with a diverse range of predicted functions (Additional file 6: Table S6). Predicted hydrolases modeled with very high confidence and alignment coverages included nucleases (TPANIC_0803 [Single-stranded DNA specific exonuclease RecJ] and TPANIC_0489 [Ribonuclease Z]), an esterase (TPANIC_0935; esterase E40), and several carbohydrate-degrading enzymes (e.g. TPANIC_0358; alpha-amylase). Transferases represented the second most abundant predicted functional class for uncharacterized *T. pallidum* proteins (22/175 [13%]) which also covered a diverse array of functions. Predicted transferases modeled with very high confidence and alignment coverages included a protein kinase (TPANIC_0307; serine/threonine kinase PrkC), a methyltransferase (TPANIC_0032; 16 s rRNA methyltransferase RsmE), and several glycosyltransferases (e.g. TPANIC_0286; Polypeptide GalNAc transferase-2) (Additional file 6: Table S6). Other functionally unannotated *T. pallidum* proteins of note that were modeled with very high confidence and coverage alignments include TPANIC_0118 (modeled against colicin Ia), TPANIC_0333 (modeled against outer membrane lipoprotein carrier protein, LolA), and five proteins (TPANIC_0548, 0856, 0858, 0859, and 0865) which were modeled on the outer membrane toluene transport protein (TbuX) from the Gram-negative opportunistic pathogen, *Ralstonia pickettii* (Additional file 6: Table S6).

When *T. pallidum* proteins of unknown function were analyzed using all high confidence tertiary structure model protein templates as an indication of function (≥90% confidence, ≥10% alignment coverage and at least one of the top 20-ranking predicted tertiary structures modeled against a protein template of known function), 167/175 (95%) uncharacterized proteins were assigned potential functions (Fig. 4b; compared to 147/175 uncharacterized proteins assigned potential functions based on the top ranking template, see above**).** The additional 20 hypothetical proteins with predicted functions are listed in Additional file 8: Table S8. These included a protein predicted to adopt a tertiary structure fold highly similar to an outer membrane deacetylase involved in facilitating the export of a biofilm adhesin polysaccharide (TPANIC_0083; Poly-beta-1,6-N-acetyl-D-glucosamine N-deacetylase, PgaB), an antibiotic resistance protein (Tpanic-0405; McbG-like protein), a cell division activator (TPANIC_0846; cell division protein, ZapA), a transcriptional activator (TPANIC_0875; transcriptional regulator, PspF), a resolvase (TPANIC_0913; Holliday junction resolvase, Hjc), and a transcription termination/anti-termination protein (TPANIC_1032; N-utilization substance G, NusG). The eight additional proteins that were modeled against structures classified by PDB as “unknown function” when all confident tertiary structure model protein template functions were taken into account are listed in Additional file 9: Table S9.

Interestingly, three *T. pallidum* proteins annotated as “hypothetical” [8] were predicted to adopt structural folds most similar to protein templates that are functionally classified by the PDB as “Blood Clotting” proteins (TPANIC_0246 and TPANIC_0750; similar to von Willebrand Factor Type A domain [vWFA]) and “Hormone/Growth factor” (TPANIC_0484; similar to Anosmin-1) (Fig. 4a and Additional file 6: Table S6). Several additional *T. pallidum* proteins of unknown function were also modeled with high confidence against other human / eukaryotic proteins, including tenascin-X, fibrinogen, and ankyrin proteins (Additional files 1, 6, 8: Tables S1, S6, S8). These findings suggest that, similar to other pathogens, molecular mimicry may contribute to *T. pallidum* pathogenesis.

### Identification of potential virulence factors

In contrast to other invasive pathogenic bacteria, genome sequencing and annotation projects have identified very few *T. pallidum* virulence factors. In light of the fact that *T. pallidum* is one of the most invasive pathogens known, we sought to use our proteome-wide tertiary structure modeling to (1) help corroborate the annotation of proteins identified as potential virulence factors from recent whole genome sequencing [8] and comparative genomics [54, 55], (2) identify putative novel virulence functions for proteins previously annotated with non-virulence related functions, and (3) identify hypothetical proteins potentially involved in *T. pallidum* pathogenesis. Therefore, we analyzed the functions of the template proteins that were used to generate the 780 high-confidence/alignment coverage *T. pallidum* tertiary structure models, and searched for protein templates that correspond to each of the 3 categories described above.

### Modeling of proteins previously annotated as putative virulence factors

As shown in Table 1 and Additional file 10: Table S10, 4/5 proteins previously annotated as putative hemolysins [8] were predicted to be most structurally similar to an *E. coli* magnesium/cobalt efflux protein (CorC). However, the same four proteins were also predicted to be structurally-similar (5th ranking template model) to a hemolysin-like protein from the non-pathogenic Gram-positive acidophilic facultative anaerobe, *Oenococcus oeni*. The remaining putative hemolysin (hemolysin III, TPANIC_1037) only exhibited confident structural homology to human adiponectin receptor protein 1 (100% confidence, 96.6% alignment coverage). These findings suggest that the five proteins are not hemolysins.

**Table 1:** Summary comparison of virulence factor candidates from whole genome sequencing and corresponding Phyre2-modeled proteins

Protein	Genome Annotation [8]	Phyre2 Model Templates: 1st rank model and potential virulence model(s)
TPANIC_0027	Putative hemolysin (HlyC)	1st rank: CorC Magnesium/Cobalt efflux protein (*E. coli*) 5th rank: Hemolysin-like protein (*Oenococcus oeni*)
TPANIC_0028	Putative hemolysin (HlyC)	1st rank: CorC Magnesium/Cobalt efflux protein (*E. coli*) 5th rank: Hemolysin-like protein (*Oenococcus oeni*)
TPANIC_0399	Type 3 (virulence-related) Secretory pathway protein (FliF)	1st rank: PrgH (*Salmonella typhimurium* type 3 secretion system protein) 2nd rank: EscJ (*E. coli* Type 3 secretion system protein)
TPANIC_0401	Type 3 (virulence-related) Secretory pathway protein (FliH)	1st rank: V-type proton ATPase subunit E (yeast) No potential virulence models
TPANIC_0402	Type 3 (virulence-related) Secretory pathway protein (FliL)	1st rank: Flagellar type 3 ATPase FliI (*Salmonella enterica*) No potential virulence models
TPANIC_0649	Putative hemolysin (TlyC)	1st rank: CorC Magnesium/Cobalt efflux protein (*E. coli*) 5th rank: Hemolysin-like protein (*Oenococcus oeni*)
TPANIC_0714	Type 3 (virulence-related) secretory pathway protein (FlhA)	1st rank: Flagellar biosynthesis protein FlhA (*Helicobacter pylori*) 3rd rank: InvA (*S. enterica* type 3 secretion system invasion protein) 5th rank: MxiA (*Shigella flexneri* type 3 secretion system invasion protein)
TPANIC_0715	Type 3 (virulence-related) secretory pathway protein (FlhB)	1st rank: YscU (*Y. pestis* type 3 secretion system protein) 2nd rank: EscU (*E. coli* type 3 secretion system protein) 3rd rank: EscU (*E. coli* type 3 secretion system protein)
TPANIC_0936	Putative hemolysin	1st rank: CorC Magnesium/Cobalt efflux protein (*E. coli*) 5th rank: Hemolysin-like protein (*Oenococcus oeni*)
TPANIC_1037	Putative hemolysin III (HlyIII)	1st rank: Human adiponectin receptor 1

Eight *T. pallidum* proteins were also previously annotated as type 3 (virulence-related) secretory pathway proteins [8]. Our tertiary-based modeling approach generated high confidence models for five of these proteins. Two of the five proteins (TPANIC_0399 and TPANIC_0715) were predicted to adopt structural folds most similar to T3SS proteins **(**Table 1 and Additional file 10: Table S10). However, the other three proteins were predicted to adopt structural folds most similar to a V-type proton ATPase protein or flagellar proteins. TPANIC_0715, but not TPANIC_0399, was also modeled with lower confidence against a flagellar biosynthesis protein **(**Additional file 10: Table S10).

A bioinformatics approach which compared the amino acid sequences of all proteins from *T. pallidum* ssp. *pallidum* and the closely related (99.8% genomic sequence identity) treponeme *T. pallidum* ssp. *pertenue* (causative agent of yaws) enabled the identification of 31 putative *T. pallidum* ssp. *pallidum* virulence factors [54, 55]. Twelve of these proteins belong to the evolutionarily distinct *Treponema pallidum* repeat (Tpr) protein family [46] and were not modeled with high confidence in the current study. Thirteen of the remaining 19 putative virulence factors were modeled with high confidence **(**Additional file 1: Table S1 and Additional file 11: Table S11). With the exception of the BamA ortholog (TPANIC_0326), TPANIC_0399 and TPANIC_0715 (see below), the potential roles of these proteins in *T. pallidum* pathogenesis remain unknown.

### Identification of potential novel virulence-related functions for annotated proteins

In the present study, three predicted *T. pallidum* protein-coding genes previously annotated with functions not directly linked to virulence were identified with high confidence by tertiary structure modeling as proteins that may play roles in *T. pallidum* pathogenesis; TPANIC_0262 (cyclic-nucleotide binding protein), TPANIC_0862 (peptidylprolyl isomerase), and TPANIC_1033 (patatin family phospholipase) (Table 2 and Additional file 12: Table S12).

**Table 2:** Genome sequencing annotated proteins with potential novel virulence-related functions identified by Phyre2 modeling

Protein	Genome Annotation	Phyre2 Models: 1st rank model and potential virulence model(s)
TPANIC_0262	Cyclic nucleotide-binding protein	1st rank: PrfA (*Listeria monocytogenes* virulence factor transcriptional regulator)
TPANIC_0862	Peptidylprolyl isomerase (FklB)	1st rank: Mip (*Legionella pneumophila* macrophage infectivity potentiator protein) 5th rank: Tcmip (*Trypanosoma cruzi* macrophage infectivity potentiator protein)
TPANIC_1033	Patatin family phospholipase	1st rank: VipD (*Legionella pneumophila* phospholipase effector protein) 2nd rank: ExoU (*Pseudomonas aeruginosa* type 3 secretion system effector protein)

Our analyses predicted TPANIC_0262 to be most structurally similar to the *Listeria monocytogenes* virulence factor transcriptional regulator, PrfA (100% confidence and 93.3% alignment coverage). This is consistent with a study that demonstrated TPANIC_0262 is a transcriptional modulator of members of the *T. pallidum* repeat (*tpr*) gene family, a group of virulence factor candidates [56]. Tertiary structure modeling identified TPANIC_0862 as a structural homolog of the essential virulence factor associated with macrophage infectivity in *Legionella pneumophila*, Mip [57, 58] (Macrophage infectivity potentiator; 100% confidence and 73.1% alignment coverage). The top two structural matches for TPANIC_1033 were cytotoxins, namely, the *L. pneumophila* phospholipase effector VipD [59] and the *Pseudomonas aeruginosa* T3SS effector ExoU [60] (both modeled at 100% confidence and 90.4% / 99.7% alignment coverages, respectively).

### Identification of hypothetical proteins with potential roles in *T. pallidum* virulence

Twenty-one of the 175 *T. pallidum* proteins of unknown function that were modeled with high confidence in the current study were predicted to exhibit high structural similarity with proteins known to be required for, or involved in, the virulence of other pathogens. These 21 *T. pallidum* proteins and their virulence model templates are summarized in Table 3 and Phyre2 modeling/virulence template details are listed in Additional file 13: Table S13.

**Table 3:** Summary of *T. pallidum* uncharacterized proteins identified by structural modeling with potential roles in virulence

Protein	Phyre2 Virulence Model Templates (% confidence / % coverage)	Roles in virulence	Expression Analyses		MSC
			cDNA/DNA	Protein	
TPANIC_0020	• TgMIC2 (*Toxoplasma gondii* micronemal protein 2 A/I domain) (98.2 / 21.2)	• Promotes active invasion [80]	2.206	E [47]	-
	• TRAP protein (*Plasmodium vivax*) (98.1 / 21.9)	• Cell adhesion & invasion [81]			
TPANIC_0126	• Outer membrane protein W (*E. coli*) (97.3 / 72.2)	• Phagocytosis resistance [82]	1.115	E [47]	+
	• Outer membrane protein A (*E. coli*) (94.8 / 64.1)	• Host adhesion, invasion and immune evasion [83]			
	• Outer membrane protein OprG (*Pseudomonas aeruginosa*) (93.9 / 70.0)	• Cytotoxicity [84]			
	• Outer membrane protein F (*P. aeruginosa*) (92.8 / 51.1)	• Host adhesion [85]			
	• NspA (Neisseria surface protein A) (90.4 / 64.1)	• Factor H-binding and complement resistance [86]			
TPANIC_0134	• Bacterial sialidases/neuraminidases (*Micromonospora viridifaciens, Vibrio cholera, Parabacteroides distasonis, Bacteroides thetaiotaomicron, Streptococcus pneumoniae*) (98.9-99.4 / 69.4-72.3)	• *V. cholera*; facilitates cholera toxin cell binding and penetration [87]	3.586	ND	+
		• *S. pneumoniae*; host cell glycan damage promotes colonization of respiratory tract [88, 89]			
TPANIC_0225	• Leucine-rich repeat surface proteins (*Eubacterium ventriosum, Faecalibacterium prausnitzii, Bacteroides* species) (99.9-100 / 72.4-83.3)	• Host adhesion [90]	2.27	ND	-
	• PcpA (*S. pneumoniae* choline-binding protein) (100 / 99.6)	• Host adhesion [91]			
TPANIC_0246	• TRAP protein (*P. vivax*) (99.9 / 35.7)	• Cell recognition & invasion [81]	0.096	E [47]	-
	• TRAP protein (*Plasmodium falciparum*) (99.8 / 35.8)	• Cell recognition & invasion [81]			
	• TgMIC2 (*T. gondii* micronemal protein 2 A/I domain) (99.8 / 30.5)	• Active invasion [80]			
TPANIC_0421	• PknD (*Mycobacterium tuberculosis* serine/threonine protein kinase, extracellular domain) (99.9 / 37.6)	• Adhesion and invasion of brain endothelia [92]	0.686	E [47]	-
TPANIC_0544	• SmcL (*Listeria ivanovii* Sphingomyelinase-C) (99.9 / 51.9)	• Cytotoxicity [93, 94]	0.811	E [47]	-
	• Beta-hemolysin toxin (*Staphylococcus aureus*) (99.9 / 51.4)	• Cytotoxicity [95]			
	• Cytolethal distending toxin protein B (*S. enterica, Haemophilus ducreyi*) (99.9 / 51.9-52.1)	• Cytotoxicity [96]			
TPANIC_0579	• YenC2 (*Yersinia entomophaga* ABC toxin; BC component) (93.8 / 36.8)	• Cytotoxicity [97]	0.268	E [47]	-
TPANIC_0594	• HP1028 (*Helicobacter* pylori lipocalin) (100 / 64.8)	• Host colonization and persistence [98, 99]	2.238	ND	+
TPANIC_0598	• BamB (*Moraxella catarrhalis* Beta barrel assembly machinery protein B) (97.9 / 56.3)	• BAM complex; assembly and insertion of beta-barrel proteins in outer membrane [100, 101]	0.272	E [47]	-
TPANIC_0625	• BamD (Beta barrel assembly machinery protein) (*Rhodothermus marinus, E. coli*) (95.9-97.0 / 27.6-46.8)	• Essential BAM complex protein; assembly and insertion of beta-barrel proteins in outer membrane [102]	0.461	E [47]	-
TPANIC_0733	• NspA (Neisseria surface protein A) (97.8 / 58.4)	• Factor H-binding and complement resistance [86]	1.735	ND	+
	• Ail (*Yersinia pestis* attachment invasion locus protein) (93.6 / 64.4)	• Host cell attachment, invasion, and complement resistance [103–105]			
TPANIC_0783	• BamB (*E. coli* Beta barrel assembly machinery protein B) (96.2 / 24.7)	• BAM complex; assembly and insertion of beta-barrel proteins in outer membrane [100, 101]	0.402	ND	-
TPANIC_0789	• LolA (*P. aeruginosa* outer- membrane lipoprotein carrier/localization protein) (99.5 / 86)	• Translocation of lipoproteins to the outer membrane [106, 107]	1.283	E [47, 48]	-
	• LprG (*M. tuberculosis* lipid- binding protein (96.8 / 71.7)	• TLR2-agonist; inhibits primary human macrophage MHC-II Ag processing [108]			
TPANIC_0854	• Bacterial sialidases/neuraminidases (*M. viridifaciens, V. cholera, P. distasonis, B. thetaiotaomicron, S. pneumoniae, P. aeruginosa*) (98.9-99.5 / 17.0-25.8)	• *V. cholera*; facilitates cholera toxin cell binding and penetration [87]	0.221	E [47]	-
		• *S. pneumoniae*; host cell glycan damage promotes colonization of upper respiratory tract [88, 89]			
TPANIC_0911	• EscU (*E. coli* type 3 secretion system protein) (100 / 96.4)	• Type 3 effector translocation into host cells [109]	0.701	ND	-
	• SpaS (*Shigella flexneri* type 3 secretion system protein) (99.9 / 94.0)	• Invasion and secretion of invasion plasmid antigens [110]			
TPANIC_0928	• SurA (*E. coli* chaperone) (100 / 84.0)	• Folding and assembly of outer membrane proteins [101]	1.049	E [47]	-
TPANIC_0966	• TolC (*E. coli* outer membrane channel protein) (100 / 79.4)	• Type 1 secretion and drug efflux [111–114]	0.856	ND	-
TPANIC_0967	• TolC (*E. coli* outer membrane channel protein) (99.0 / 85.7)	• Type 1 secretion and drug efflux [111–114]	2.844	E [47]	+
TPANIC_0968	• TolC (*E. coli* outer membrane channel protein) (98.8 / 85.2)	• Type 1 secretion and drug efflux [111–114]	3.256	E [47]	+
TPANIC_0969	• TolC (*E. coli* outer membrane channel protein) (99.0 / 87.0)	• Type 1 secretion and drug efflux [111–114]	2.751	E [47, 48]	+

cDNA/DNA ratios indicate transcript expression levels from a previous rabbit infection study where a value of 1.0 represents the mean transcript expression level for all *T. pallidum* genes in the study [45]. E; proteins known to be expressed during rabbit infection (ND; proteins not detected) [47, 48]. MSC; *T. pallidum* subspecies *pallidum* proteins that contain major sequence changes compared to orthologs from subspecies *pertenue* and *T. paraluiscuniculi* [55, 61]

Seven of the 21 *T. pallidum* hypothetical proteins were predicted to be structural orthologs of virulence factors that are involved in mediating host adhesion and invasion (TPANIC_0020, TPANIC_0126, TPANIC_0225, TPANIC_0246, TPANIC_0421, TPANIC_0733, and TPANIC_0911). Four proteins exhibited high structural homology to orthologs involved in type 1 secretion and drug efflux (TPANIC_0966–0969). Phyre2 also predicted high structural similarity of three hypothetical proteins to immune modulators (TPANIC_0126, TPANIC_0733, and TPANIC_0789) and cytotoxins (TPANIC_0126, TPANIC_0544, and TPANIC_0579). Two proteins were modeled on sialidases (TPANIC_0134 and TPANIC_0854), and one on a protein that functions in host colonization and persistence (TPANIC_0594). Five of the 21 proteins were modeled against proteins involved in the folding, assembly, translocation, and insertion of proteins in the outer membrane (TPANIC_0598, TPANIC_0625, TPANIC_0783, TPANIC_0789, and TPANIC_0928).

DNA microarray-based analysis of the *T. pallidum* transcriptome following experimental rabbit infection showed that the genes encoding 11 of the 21 uncharacterized proteins identified as virulence factor candidates in the current study were all expressed at higher levels (cDNA/DNA ratio > 1.0) than the mean transcript level (cDNA/DNA ratio = 1.0) of all *T. pallidum* genes in the array [45] (Table 3). In fact, eight of these potential virulence factor genes were expressed at approximately two- to four-fold higher levels than the mean *T. pallidum* transcript level whereas four of the 21 potential virulence factor genes were identified as being weakly expressed during infection (cDNA/DNA ratio ≤ 0.36) [45] (Table 3). Furthermore, mass spectrometry-based proteomics studies have also detected peptides from 14 of the 21 uncharacterized proteins identified as putative virulence factors, demonstrating that at least two thirds of these proteins are expressed during rabbit infection [47, 48] (Table 3). Interestingly, 7/21 (33%) hypothetical proteins identified with potential roles in *T. pallidum* virulence were also previously shown to be among the set of orthologous proteins from the closely-related treponemes, *T. pallidum* ssp. *pertenue* and *T. paraluiscuniculi*, that contain major sequence changes (MSC) (Table 3) [55, 61]. In contrast, only 89 proteins from the whole *T. pallidum* proteome (9%) were found to contain major sequence differences when compared to the closely-related treponemal orthologs (*p* = 0.002; Fisher’s exact test) [62].

## Discussion

Using a computational modeling-based approach, we generated high-confidence tertiary structure models and inferred functional annotations for 80% of the *T. pallidum* proteome. Out of the 196 proteins modeled with low confidence (20% of the *T. pallidum* proteome), 150 proteins were functionally-unannotated in the published *T. pallidum* proteome, [8]. The high number of functionally-unannotated proteins modeled with low confidence further highlights the phylogenetic divergence of *T. pallidum*. Members of this structurally-divergent and functionally-uncharacterized protein subset may contribute to the unusual structural and virulence properties of *T. pallidum*. In contrast, almost a quarter of the proteins that were modeled with low-confidence had previously been assigned specific functions in the published *T. pallidum* proteome [8]**.** The physicochemical properties of this group of proteins varied widely. In addition, the average values corresponding to some of the physicochemical properties were similar to those previously reported for all *T. pallidum* proteins [7], including average molecular mass and isoelectric point. Therefore, unusual physicochemical characteristics cannot be correlated with the failure to generate high-confidence models for this group of proteins. Several of the proteins in this group belong to the phylogenetically distinct Tpr family of proteins [46], which may partially explain why almost a quarter of these proteins were not modeled with high confidence. Subcellular localization analyses predicted half of the proteins to be localized to the inner membrane. However, many high-confidence tertiary structure models of known and predicted inner membrane proteins were generated in the current study, indicating this predicted subcellular localization bias is also unlikely to explain the inability to generate high-confidence models for this group of 46 proteins that have been previously annotated using genome sequencing/annotation pipelines. The most likely explanation why these 46 proteins were annotated based on primary structure but not by tertiary structure modeling is that the proteins share some degree of amino acid sequence homology with other annotated proteins, however high resolution structures of these proteins have not been deposited in the PDB.

Importantly, our tertiary structure modeling pipeline was able to predict the same function as primary structure-based annotations from genome sequencing/annotation pipelines for almost 90% of compared proteins. In addition, we observed a high degree of correlation at both the structural and functional levels when we compared our molecular modeling-based protein function predictions with the functions reported for 11 *T. pallidum* proteins that were assigned specific functions using structure-to-function experimental approaches. Together, these results (1) suggest our structural bioinformatics pipeline is a promising approach for assigning tentative protein functions based on tertiary structural modeling on a proteome-wide level, and (2) strengthen the published primary structure-based annotation of more than half of all *T. pallidum* proteins. This latter finding suggests this approach could be used as a complementary bioinformatics tool to corroborate primary structure-based methodologies in genome annotation pipelines. Furthermore, confident tertiary structure modeling-based annotation of proteins that are found to have different functional predictions from primary structure homology-based annotations could help identify potential misannotated proteins in genome sequencing pipelines.

*Treponema pallidum* is a phylogenetically distinct bacterium, highlighted by the fact that one third (327/978) of predicted protein-coding genes have no known orthologs and/or assigned function [8]. Furthermore, at 1.14 Mb, *T. pallidum* has the smallest spirochetal genome and one of the smallest bacterial genomes sequenced to date [7]. Obligate pathogenic bacteria, such as *T. pallidum*, often undergo genomic reduction characterized by the loss of non-essential genes as an evolutionary adaptation mechanism for the rich and complex host environment that is encountered during infection [63, 64]. Thus, it is likely that the *T. pallidum* proteins of unknown function play important roles in the biology of this treponeme, and may also contribute to its unique structural and virulence properties.

In the current study, 175 of the 327 *T. pallidum* proteins with no known orthologs and/or assigned function [8] were modeled with high confidence/alignment coverage. Of these 175 proteins, 167 (95%) were assigned predicted biological roles based on the function of the corresponding model templates. Importantly, previous expression-profiling –omics studies [45, 47, 48] have also demonstrated that (1) 99% of the 175 genes predicted to encode *T. pallidum* proteins of unknown function, including the majority that are annotated as “hypothetical proteins”, represent functional genes, and (2) at least 61% of these functionally-uncharacterized *T. pallidum* proteins are expressed during infection.

Most of the 175 *T. pallidum* proteins of unknown function were modeled against hydrolases and transferases using PDB functional classifications. Interestingly, three “hypothetical” *T. pallidum* proteins [8] were also predicted to adopt structural folds similar to “Blood Clotting” proteins (TPANIC_0246 and TPANIC_0750) and a “Hormone/Growth factor” (TPANIC_0484). Specifically, Phyre2 predicted structural similarity of TPANIC_0246 and TPANIC_0750 to von Willebrand Factor Type A domain (vWFA), the prototype domain of the vWF domain-containing protein superfamily [65]. The TPANIC_0750 result is in agreement with previous molecular modeling that also predicted similarity to vWFA [53]. The tertiary structure of TPANIC_0484 was predicted to be most similar to Human Anosmin-1, a fibronectin type 3 domain-containing extracellular matrix protein involved in nerve cell migration, axon outgrowth, and nerve cell adhesion [66, 67]. The finding that a subset of *T. pallidum* proteins of unknown function is predicted to adopt structural folds that closely resemble host proteins suggests that *T. pallidum* may use molecular mimicry as a pathogenic strategy for the subversion and exploitation of normal host cellular functions during infection.

It has been demonstrated, using high-resolution microscopy techniques, that *T. pallidum* contains few outer membrane proteins [9, 10]. Interestingly, our analyses identified five functionally-unannotated *T. pallidum* proteins (TPANIC_0548, 0856, 0858, 0859, and 0865) which were modeled on the toluene transport protein (TbuX) from the Gram-negative opportunistic pathogen, *Ralstonia pickettii*. This 14-stranded beta barrel-containing outer membrane protein belongs to the FadL fatty acid transporter family [68, 69] and functions in the uptake and degradation of aromatic hydrocarbons, including toluene [70, 71]. Given that *T. pallidum* is unlikely to encounter aromatic hydrocarbons during infection and that aromatic hydrocarbon degradation pathways have not been identified in the *T. pallidum* genome [7, 8], it is likely that the substrate specificities of the five potential *T. pallidum* outer membrane transport proteins differ from TbuX. In agreement with a previous molecular modeling study [72], these five *T. pallidum* proteins were also modeled on FadL from *E. coli* and *P. aeruginosa* with similar high confidence and alignment coverage scores as those observed for the top ranking model template, TbuX. *Treponema pallidum* has very limited biosynthetic capabilities, including its inability to synthesize fatty acids de novo, and must therefore rely on the host for nutrients [7]. Thus, these findings suggest that the five potential *T. pallidum* transport proteins are more likely to be involved in the transport of hydrophobic molecules such as fatty acids across the outer membrane during infection.

Genome sequencing and annotation projects have identified very few *T. pallidum* proteins that are potential orthologs of known virulence factors from other pathogenic bacteria [7, 8]. This finding is surprising as *T. pallidum* is a strict pathogen capable of infecting every tissue and organ, and is one of the most invasive pathogens known. Five potential hemolysins were identified in *T. pallidum* genome sequencing pipelines [7, 8], however tertiary structure modeling suggests that these proteins are unlikely to function as toxins which is consistent with previous results that failed to show hemolytic activity for recombinant forms of these proteins [62, 73]. A further eight *T. pallidum* proteins have been annotated in the Nichols strain as T3SS proteins [8]. Five of these proteins were modeled with high confidence/alignment coverage in the present study, two of which were also predicted to adopt structural folds similar to T3SS proteins. Two evolutionarily-related, but functionally-distinct, forms of T3SS are often found in Gram-negative bacteria that are responsible for mediating translocation of proteins across the inner and outer membranes: (1) the flagellar T3SS that mediates transport and export of proteins that comprise the flagellar filament structure used for motility and (2) the non-flagellar T3SS (also known as the injectisome) that secretes effectors into host cells [74, 75]. These two T3SSs are comprised of several orthologous proteins and also share several ultrastructural similarities [76]. *Treponema pallidum* possesses flagellar filaments that are located entirely within the periplasmic space between the inner and outer membranes [9, 10]. However, to date no evidence of an injectisome or needle-like T3SS structure has been detected on the surface of *T. pallidum,* including through the use of high-resolution cryo electron tomography [9, 10]. In addition, several protein structures from both the flagellar and non-flagellar/injectisome T3SSs remain to be solved and deposited in the PDB. Therefore, it is possible that proteins modeled by Phyre2 as injectisome-like T3SS-associated proteins actually belong to the flagellar T3SS subtype. Interestingly, examples of T3SSs have been reported that do not conform fully to either subtype. For example, it has been reported that flagellar T3SS of some pathogenic bacteria have also evolved the ability to secrete virulence factors [77–79]. Thus, another possibility is that the flagellar T3SS of *T. pallidum* has also evolved the ability to transport, and possibly export, non-flagellar filament proteins. This hybrid export system would be consistent with the highly invasive nature of *T. pallidum* and its obligate pathogenic lifestyle and resulting reduced genome. If this system can indeed function as a T3SS, the mechanism of effector export and the export machinery would be predicted to differ greatly from the injectisome needle complexes found in conventional Gram-negative pathogens, such as *Salmonella typhimurium*, owing to the phylogenetic divergence of this pathogen and its unusual subcellular flagella location.

Twenty-one *T. pallidum* proteins of unknown function were predicted to exhibit structural similarity with proteins experimentally confirmed to be required for virulence in other pathogens. These proteins were modeled on virulence factors with roles in host adhesion and invasion, type 1 secretion and drug efflux, immune modulation, cytotoxicity, sialidase activity, host colonization and persistence, and five proteins that function in the folding, assembly, translocation, and insertion of proteins in the outer membrane. Although the protein templates used to model these latter five proteins are normally located within the periplasm of other bacteria and do not mediate direct interactions with the host, they are important for outer membrane protein localization and surface exposure, which is an essential mechanism for facilitating host-pathogen interactions during infection. Importantly, some of our tertiary structure modeling findings correlated with an alternative molecular modeling approach used by Radolf and Kumar [72]. Specifically, three potential virulence factors identified in the current work (Tp0733, Tp0966 and Tp0967) were modeled in both studies against protein templates known to adopt beta barrel folds, that localize to the outer membrane, and that function in complement resistance and drug efflux. In the absence of high sequence identity, these findings have tentatively identified several uncharacterized proteins that, based on high-confidence structural modeling predictions, may represent important mediators of *T. pallidum* virulence.

The clinical manifestations of infections with *T. pallidum* subspecies *pallidum* and the less-invasive subspecies *pertenue* are readily distinguished. However, a comparative genomics study demonstrated identical gene synteny and 99.8% genetic identity between the genomes [55]. Similarly, rabbit venereal spirochetosis-causing *Treponema paraluiscuniculi* shares greater than 99% sequence identity in conserved regions and identical gene synteny with *T. pallidum* [61]. However, *T. paraluiscuniculi* is non-infectious in humans and rabbit infection is also characterized by different and less-invasive disease symptoms. Orthologous proteins from these closely-related pathogens that contain at least one MSC may therefore represent potential treponemal virulence factors that could account for differences in host specificity and clinical manifestations [55, 61]. Out of the 21 *T. pallidum* putative virulence factors identified in the current study, seven (33%) were previously shown to contain at least one MSC when compared with the corresponding orthologous proteins in the two closely-related treponemes described above [55, 61]. However, a total of only 89 MSC-containing proteins were identified in the whole *T. pallidum* proteome (9%) [62]. The high proportion of hypothetical proteins with potential virulence roles that also harbour major primary structure differences compared to their orthologs in less-invasive subspecies and *T. paraluiscuniculi* is consistent with our tertiary structure modeling findings that suggest roles for this subset of proteins in the pathogenesis of *T. pallidum* subspecies *pallidum*.

Here we employed a high throughput structural bioinformatics approach for the prediction of protein function from tertiary structure modeling on a proteome-wide scale, often in the absence of significant amino acid sequence homology. However, as with other methods for predicting protein function, there are limitations associated with this approach. First, high confidence tertiary structure modeling relies entirely on the deposition of high-resolution protein structures in the PDB, that through structural similarities serve as protein templates for predicting the three dimensional fold of the query protein. If these structural templates have not been solved and/or deposited in the PDB, then tertiary structure modeling and function prediction is not possible. Second, assignment of predicted protein functions is complicated when the query protein (or a specific region of the query protein) is modeled against several functionally diverse protein templates which all have similar high confidence and alignment coverage scores. In this scenario, the amino acid identities of the query sequence and protein templates may be taken into account to identify any template(s) with significantly higher amino acid homology as increasing identity often correlates with an increased likelihood of related function. Third, predicting functions for protein structures modeled with high confidence but low alignment coverage increases the risk of misannotation of protein function. For example, the function of the template used to model a single domain or isolated region that corresponds to only 10–20% of amino acids in the query protein sequence may not be representative of the overall/multiple functions of the full-length query protein. However, in the present study only 5% of all high confidence (≥90%) models were based on alignment coverages of 20% or less. Conversely, almost three quarters of all *T. pallidum* proteins were confidently modeled with at least 50% coverage and over half of all *T. pallidum* proteins were confidently modeled with at least 80% alignment coverage. Thus, the risk of functional annotation errors for *T. pallidum* proteins due to low sequence coverage in the current study is very low. However, it is important to note that tertiary structure model alignment coverage refers only to the percentage of amino acids in the query protein sequence that align with the template protein, but does not provide any information regarding the size or percentage of amino acids of the model template protein that was used to generate the alignment. Therefore, caution must be taken in assigning function to a query protein based on a tertiary structure model template that contains additional functionally-characterized domains that may have been used to annotate the function of the template protein during the genome sequencing and annotation pipeline. Finally, it should be noted that this molecular modeling-based annotation approach has assigned protein functions in the absence of experimental validation, and that these putative functions can only be truly validated experimentally.

## Conclusions

In the present study, we used the tertiary structure modeling server, Phyre2, to investigate the potential function of all 978 predicted proteins from the causative agent of syphilis, *Treponema pallidum* subspecies *pallidum* (Nichols strain). This structural modeling-based approach provided insight into the putative functions of approximately 80% of *T. pallidum* proteins. Furthermore, functional predictions inferred from proteome-wide structural modeling corroborated the amino acid-based annotation of over half of all *T. pallidum* proteins, suggesting that this methodology is useful for increasing confidence in the annotated predicted protein functions derived from genome sequencing projects. Importantly, our tertiary structure modeling approach was also able to predict structural models based on functionally-annotated templates for over half of all uncharacterized *T. pallidum* proteins, thereby providing novel insight into this enigmatic group of proteins that may have potential roles in the unusual ultrastructure and unique pathogenesis of *T. pallidum*. This suggests our approach has the potential for enhancing understanding of protein function across all taxonomic groups and may be particularly applicable for the functional annotation of phylogenetically distinct organisms. This is the first report of a proteome-wide structural modeling approach that has provided further insight into the potential functions of the majority of *T. pallidum* proteins. In light of the dynamic nature of the protein databank and continued technological advances in next-generation sequencing that facilitate the improved sequencing accuracy and annotation of whole genomes, regular re-analyses of whole proteomes using molecular modeling will continue to improve upon our understanding of structure-function relationships and is likely to provide further insight into the molecular mechanisms underlying *T. pallidum* pathogenesis.

## Additional files

Additional file 1: **Table S1 (Part 1).** Raw unedited Phyre2 data corresponding to 19,560 templates used for *T. pallidum* whole proteome modeling. (XLSX) **Part 2.** Raw unedited Phyre2 data corresponding to 780 *T. pallidum* proteins modeled with high confidence (≥90% confidence; ≥10 alignment coverage). (XLSX 3537 kb)

Additional file 2: **Table S2.** *T. pallidum* proteins (*N* = 150) modeled by Phyre2 with confidence scores below 90% and annotated in the published *T. pallidum* proteome as hypothetical/putative membrane proteins. (XLSX 34 kb)

Additional file 3: **Table S3.** *T. pallidum* proteins (*N* = 46) modeled by Phyre2 with confidence scores below 90% with functional annotations in the published proteome. (XLSX 25 kb)

Additional file 4: **Table S4.** Comparison of *T. pallidum* primary and tertiary structure-based functional annotations using only the top-ranked Phyre2 model template for each protein comparison. (XLSX 33 kb)

Additional file 5: **Table S5.** Comparison of *T. pallidum* primary and tertiary structure-based functional annotations using all confident Phyre2 model templates for each protein comparison. (XLSX 34 kb)

Additional file 6: **Table S6.** Top-ranking Phyre2 model templates corresponding to all functionally-unannotated *T. pallidum* proteins (*N* = 175). (XLSX 40 kb)

Additional file 7: **Table S7.** Functionally-unannotated *T. pallidum* proteins (*N* = 28) with top-ranking templates modeled against structures classified by the PDB as “unknown function”. (XLSX 13 kb)

Additional file 8: **Table S8.** Functionally-unannotated *T. pallidum* proteins [*N* = 20] with Phyre2-based predicted functions using all confident model templates. (XLSX 30 kb)

Additional file 9: **Table S9.** Functionally-unannotated *T. pallidum* proteins [*N* = 8] modeled exclusively against PDB structures of unknown function. (XLSX 14 kb)

Additional file 10: **Table S10.** Phyre2 Modeling of *T. pallidum* proteins previously annotated as putative virulence factors. (XLSX 17 kb)

Additional file 11: **Table S11.** Comparison of potential virulence factors identified by comparative genomics and Phyre2 modeling. (DOCX 13 kb)

Additional file 12: **Table S12.** Genome-annotated *T. pallidum* proteins with potential novel virulence-related functions identified by Phyre2 modeling. (XLSX 11 kb)

Additional file 13: **Table S13.** Proteins of unknown function with potential roles in *T. pallidum* virulence. (XLSX 21 kb)

## Abbreviations

MSA: Multiple sequence alignment
MSC: Major sequence changes
PDB: Protein data bank
T3SS: Type 3 secretion system
*Tp*: *Treponema pallidum*
TPANIC: *Treponema pallidum* Nichols strain (nomenclature used to annotate proteins)
