# Supplementary material for: Functional insights from proteome-wide structural modeling of Treponema pallidum subspecies pallidum, the causative agent of syphilis
Source: BMC Struct Biol. 2018 May 16;18:7. doi: 10.1186/s12900-018-0086-3 (PMC5956850; doi:10.1186/s12900-018-0086-3)
Supplement: Supplementary file 11 — Table S11. Comparison of potential virulence factors identified by comparative genomics and Phyre2 modeling. (DOCX 13 kb) [file 12900_2018_86_MOESM11_ESM.docx]

**Table S11. Comparison of Potential Virulence Factors Identified by Comparative Genomics and Phyre2 Modeling.**

| ***T. pallidum* Protein** | **Genome Annotated Function** | **Top Ranking Phyre2 Model Template** |
| --- | --- | --- |
| Tpanic_0155 | M23B subfamily peptidase | M23 peptidase domain protein (*Neisseria meningitidis*) |
| Tpanic_0171 | 15 kDa lipoprotein, Tpp15 | Uncharacterized protein Cpe2226 (*Clostridium perfringens*) |
| Tpanic_0326 | BamA | Outer membrane protein assembly factor BamA (*Neisseria gonorrhoeae*) |
| Tpanic_0399 | IIISP family Type III virulence-related secretory pathway protein | Prgh (Salmonella typhimurium type III secretion system protein) |
| Tpanic_0401 | IIISP family Type III virulence-related secretory pathway protein | V-type proton ATPase subunit E (yeast) |
| Tpanic_0402 | IIISP family Type III virulence-related secretory pathway protein | Flagellar type III atpase FliI (*Salmonella enterica*) |
| Tpanic_0572 | putative membrane protein | Uncharacterized protein Cpe2226 (*Clostridium perfringens*) |
| Tpanic_0574 | lipoprotein antigen Tp47 | Tp47, 47 kda membrane antigen (*T. pallidum*) |
| Tpanic_0680 | O-sialoglycoprotein endopeptidase | O-sialoglycoprotein endopeptidase/protein kinase (*Methanococcus jannaschii*) |
| Tpanic_0714 | IIISP family Type III virulence-related secretory pathway protein | Flagellar biosynthesis protein FlhA (*Helicobacter pylori*) |
| Tpanic_0715 | IIISP family Type III virulence-related secretory pathway protein | YscU (*Y. pestis* type III secretion system protein) |
| Tpanic_0971 | Tp34 lipoprotein | Tp34, 34 kda membrane antigen (*T. pallidum)* |
| Tpanic_1038 | Bacterioferrin | Dps-1, DNA-binding stress response protein (*Deinococcus radiodurans*) (#1)  TpF1 antigen (*T. pallidum*) (#2) |
